# Supplementary material for: Inefficient Transmission of African Swine Fever Virus to Sentinel Pigs from an Environment Contaminated by ASFV-Infected Pigs under Experimental Conditions
Source: Transbound Emerg Dis. 2024 Jan 18;2024:8863641. doi: 10.1155/2024/8863641 (PMC12017190; doi:10.1155/2024/8863641)
Supplement: Supplementary Materials — Figure S1: detection of ASFV DNA on spiked surfaces using different swabs and elution media. Figure S2: detection of infectious ASFV in nasal swabs from inoculated animals in Experiment 2. [file 8863641.f1.pdf]

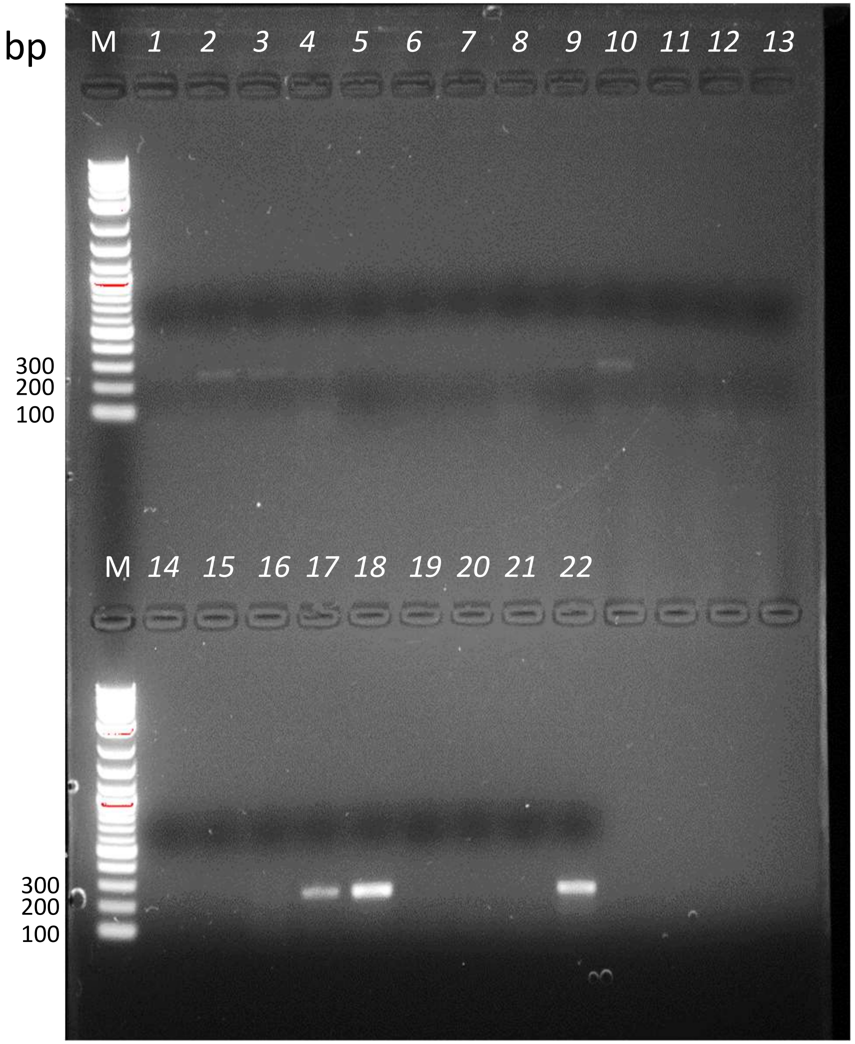

M	Molecular weight marker*
1	10^6 TCID$_{50}$/ml dust swab PBS
2	10^6 TCID$_{50}$/ml cotton swab PBS
3	10^6 TCID$_{50}$/ml swab straw PBS
4	10^6 TCID$_{50}$/ml straw PBS
5	10^5 TCID$_{50}$/ml dust swab PBS
6	10^5 TCID$_{50}$/ml cotton swab PBS
7	10^5 TCID$_{50}$/ml swab straw PBS
8	10^5 TCID$_{50}$/ml straw PBS
9	10^6 TCID$_{50}$/ml dust swab medium
10	10^6 TCID$_{50}$/ml cotton swab medium
11	10^6 TCID$_{50}$/ml straw swab medium
12	10^6 TCID$_{50}$/ml straw medium
13	10^5 TCID$_{50}$/ml swab medium
14	10^5 TCID$_{50}$/ml cotton swab medium
15	10^5 TCID$_{50}$/ml straw swab medium
16	10^5 TCID$_{50}$/ml straw medium
17	10^5 TCID$_{50}$/ml original virus dilution
18	10^6 TCID$_{50}$/ml original virus dilution
19	Control PBS
20	Control medium
21	No template control (2µl H20)
22	Positive control DNA

*Quick load 2-Log DNA Ladder (New England Biolabs)

Figure S1: Detection of ASFV DNA on spiked surfaces using different swabs and elution media. ASFV suspensions (50 µl) with different viral concentrations (10^5, 10^6 TCID$_{50}$/ml) were smeared onto petri dishes or dropped on straw and swabbed with either cotton or dust swabs. The swabs and straw were immersed in PBS or culture medium to elute the virus. ASFV DNA was detected by PCR after nucleic acids extraction from the eluate volumes.

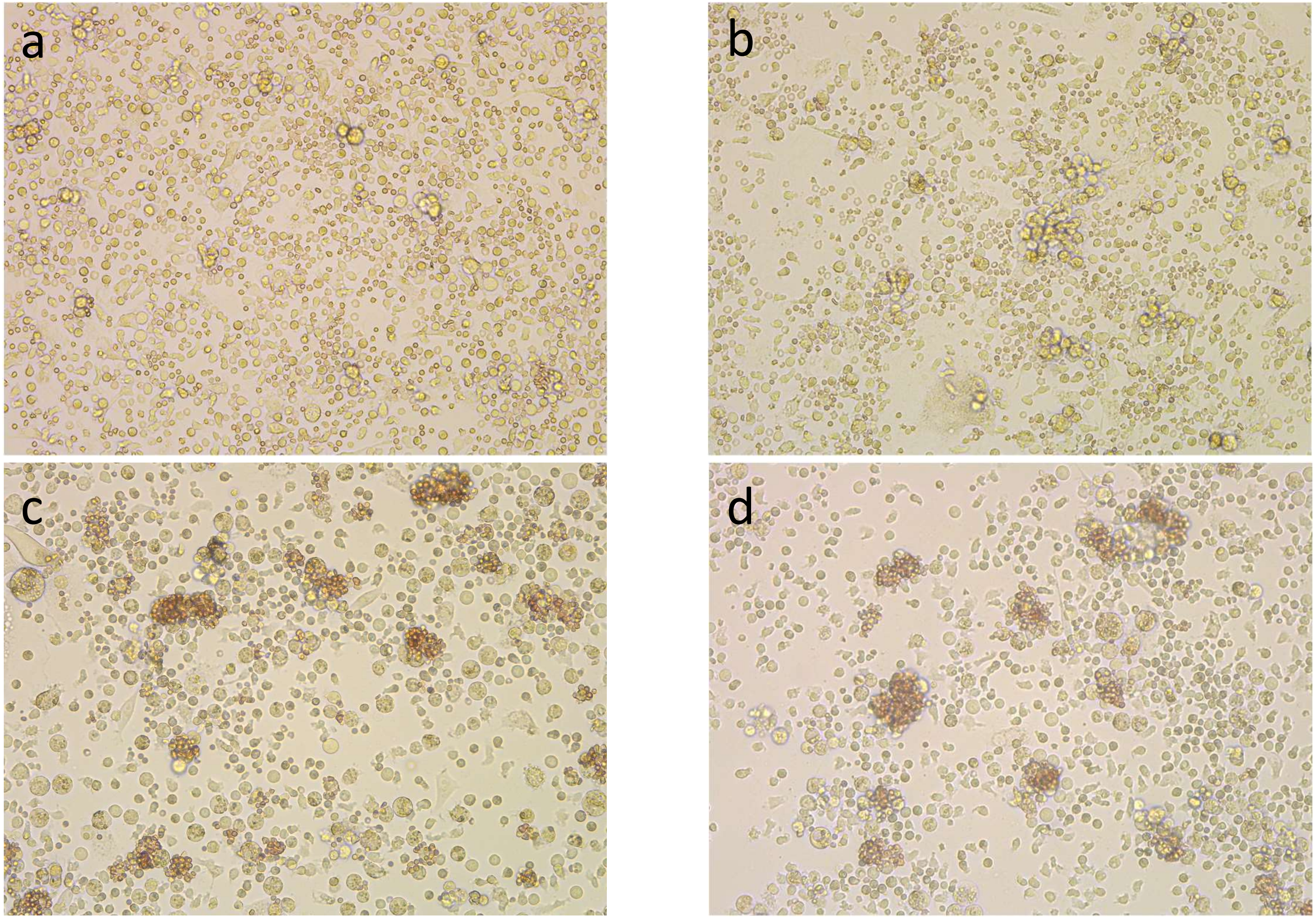

Figure S2: Detection of infectious ASFV in nasal swabs from inoculated animals in Experiment 2. Porcine bone marrow primary cell cultures were inoculated with nasal swab elution solutions and observed under the light microscope for up to 6 days for development of hemadsorption rosettes (red blood cells that are naturally present in the cultures). Panels are representative of negative (a-b) and positive (c-d) results for the presence of virus. (a) Mock inoculated culture. (b) Nasal swab from Pig 1 at 5 dpi. (c) Nasal swab from Pig 3 at 3 dpi. (d) Nasal swab from Pig 2 at 5 dpi.
